# An EST-SSR based genetic linkage map and identification of QTLs for anthracnose disease resistance in water yam (*Dioscorea alata* L.)

**DOI:** 10.1371/journal.pone.0197717

**Published:** 2018-10-10

**Authors:** Ranjana Bhattacharjee, Christian O. Nwadili, Christopher A. Saski, Agre Paterne, Brian E. Scheffler, Joao Augusto, Antonio Lopez-Montes, Joseph T. Onyeka, P. Lava Kumar, Ranajit Bandyopadhyay

**Affiliations:** 1 International Institute of Tropical Agriculture (IITA), Ibadan, Nigeria; 2 Michael Okpara University of Agriculture, Umudike, Abia state, Nigeria; 3 National Root Crops Research Institute, Umudike, Umuahia, Abia State, Nigeria; 4 Institute for Translational Genomics, Genomics and Computational Biology Laboratory, Clemson University, Clemson, South Carolina, United States of America; 5 Genomics and Bioinformatics Research Unit, USDA-ARS, Stoneville, Mississippi, United States of America; CIRAD, FRANCE

## Abstract

Water yam (*Dioscorea alata* L.) is one of the most important food yams with wide geographical distribution in the tropics. One of the major constraints to water yam production is anthracnose disease caused by a fungus, *Colletotrichum gloeosporioides* (Penz.). There are no economically feasible solutions as chemical sprays or cultural practices, such as crop rotation are seldom convenient for smallholder farmers for sustainable control of the disease. Breeding for development of durable genetic resistant varieties is known to offer lasting solution to control endemic disease threats to crop production. However, breeding for resistance to anthracnose has been slow considering the biological constraints related to the heterozygous and vegetative propagation of the crop. The development of saturated linkage maps with high marker density, such as SSRs, followed by identification of QTLs can accelerate the speed and precision of resistance breeding in water yam. In a previous study, a total of 1,152 EST-SSRs were developed from >40,000 EST-sequences generated from two *D*. *alata* genotypes. A set of 380 EST-SSRs were validated as polymorphic when tested on two diverse parents targeted for anthracnose disease and were used to generate a saturated linkage map. Majority of the SSRs (60.2%) showed Mendelian segregation pattern and had no effect on the construction of linkage map. All 380 EST-SSRs were mapped into 20 linkage groups, and covered a total length of 3229.5 cM. Majority of the markers were mapped on linkage group 1 (LG 1) comprising of 97 EST-SSRs. This is the first genetic linkage map of water yam constructed using EST-SSRs. QTL localization was based on phenotypic data collected over a 3-year period of inoculating the mapping population with the most virulent strain of *C*. *gloeosporioides* from West Africa. Based on threshold LOD scores, one QTL was consistently observed on LG 14 in all the three years and average score data. This QTL was found at position interval of 71.1–84.8 cM explaining 68.5% of the total phenotypic variation in the average score data. The high marker density allowed identification of QTLs and association for anthracnose disease, which could be validated in other mapping populations and used in marker-assisted breeding in *D*. *alata* improvement programmes.

## Introduction

Yams (*Dioscorea* spp.) are among the primary food crops, and rank second after cassava, in West Africa. More than 54 million tons of yams are produced in sub-Saharan Africa annually on 4.6 m ha. Over 95% of this production comes from the “yam belt” of West Africa that includes Nigeria, Benin, Togo, Ghana, and Côte d’Ivoire [1]. Although yam production in Africa is 40% that of cassava, the value of yam production exceeds all other African staple crops and is equivalent to the summed value for the top three cereal crops (maize + rice + sorghum) (http://www.iita.org). Yam tubers are the preferred staple food in the region considering its nutritional qualities such as carbohydrates, vitamin C, essential minerals and dietary fiber. The consumer demand for yams in West Africa is very high but its productivity is declining in the region due to several biotic and abiotic constraints.

*Dioscorea alata* L. (known as water yam or greater yam) is one of the major cultivated species with wide geographical distribution including West Africa, Asia, Latin America and the Pacific [2]. It is superior to most cultivated yam species because of its genetic potential to yield even under low to average soil fertility, ease of propagation through production of bulbils and high multiplication ratio, early vigor for weed suppression, and low post-harvest losses [3]. It is a dioecious species with ploidy level ranging from 2n = 2x = 40 to 2n = 4x = 80 [4] with basic chromosome number of x = 20. Cultivars of *D*. *alata* are exclusively clonally propagated using small tubers or small pieces of tubers (mini-setts) that excludes sexual reproduction and represent a constraint for adaptation to biotic and abiotic stresses. The most serious biotic stress of *D*. *alata* is anthracnose disease caused by an airborne fungal pathogen *Colletotrichum gloeosporioides* (Penz) [5, 6]. The disease causes mild to acute leaf necrosis and shoot die-back resulting in yield losses of up to 90% during severe conditions [7, 8]. The disease is also considered to be responsible for the disappearance of some of the popular cultivars of *D*. *alata* in the Caribbean. Several studies focusing on biodiversity of *C*. *gloeosporioides* that causes anthracnose disease on yam across different geographical locations including the French West Indies [9, 10], Nigeria [11, 5] and West Africa [12] revealed high genetic and pathogenic variance among the isolates. This high genetic and pathogenic variation in the populations of *C*. *gloeosporioides* suggests that there is high likelihood for the breakdown of existing resistance and development of new strains of the pathogen [13, 14, 15]. Screening exotic germplasm accessions and selecting for new sources of durable disease resistance is the most reliable approach to long-term management of this disease. However, it is challenging to adequately evaluate yam genotypes for anthracnose disease response under multi-location field conditions because of the high variation among the pathogen isolates and variability in symptoms development across seasons [14, 15]. Furthermore, the standardization of screening protocol for anthracnose disease scoring is underway (Kumar et al., unpublished), although disease screening in controlled environments is favorable for both disease development and plant growth [16, 17].

The genetic improvement of water yam at International Institute of Tropical Agriculture (IITA), Nigeria; Center for Tropical Crops Research Institute (CTCRI), India; Centre de recherche Antilles (INRA), Guadeloupe; and Centre de Cooperation Internationale en Recherche Agronomique pour le Développement (CIRAD), Guadeloupe; are in progress to develop high-yielding anthracnose resistant varieties through classical breeding using phenotypic observations. It is however very difficult to breed for resistant varieties using conventional methods due to several constraints related to the nature of the crop such as long growth cycle (9–11 months), dioecious, poor to erratic flowering, polyploidy, vegetative propagation and heterozygous genetic makeup [18, 19]. The use of molecular techniques, such as the candidate gene approach to dissect the underlying gene (s) for the trait of interest, would accelerate efforts in introgressing disease resistance into preferred genetic background. Earlier studies on anthracnose disease in water yam showed that resistance is likely to be dominant and quantitatively inherited [16, 20]. Mignouna et al [19] used RAPD markers to demonstrate that there is a single major dominant locus, designated as *Dcg*-1, controlling resistance in the breeding line, TDa 95/00328 and this resistance is isolate specific. Moreover, it was also suggested that combining both conventional and molecular approaches would be the best approach to develop varieties with a wide range of stable resistant gene (s) to sustain against a broad spectrum of fungal pathogens for yam improvement. It is an already well-established fact that speed, economics, and precision can be improved in breeding cycles by using molecular markers and dense genetic linkage maps. The availability of molecular markers and linkage maps allows identification of locations or markers linked with the trait of interest, thus making it possible to manipulate and understand the inheritance of quantitative traits such as disease resistance.

Attempts have been made to construct linkage maps in *Dioscorea* spp. and the first map was developed using AFLP and sequence-tagged microsatellite markers in dioecious diploid wild species *D*. *tokoro* [21]. Similarly, the RAPD and AFLP-based genetic maps were developed in *D*. *alata* by Mignouna et al [19, 22] and Petro et al [20], respectively. Similarly, integrated genetic and physical map of *D*. *rotundata* was developed by anchoring the scaffold sequences to restriction site associated DNA (RAD)-based genetic map resulting in 21 chromosome-scale pseudo-molecules or linkage groups [23]. The sequence-tagged microsatellite markers developed in *D*. *tokoro* [21] were not suitable for application in cultivated species such as *D*. *rotundata* and *D*. *alata*. The efforts on development of linkage maps for QTL detection in *D*. *alata* has been limited due to unavailability of enough molecular markers that are efficient, user-friendly, and co-dominant such as simple sequence repeat (SSRs) markers or single nucleotide polymorphism (SNPs) markers. Subsequently, a total of 44,757 EST-sequences, with an average length of 500 bp, were generated from the cDNA libraries of two resistant (TDa 87–01091 and TDa 95/00328) and one susceptible (TDa 95–310) genotypes of *D*. *alata* for anthracnose disease [24]. These EST-sequences were further annotated to obtain 1,152 EST-SSRs [25] and a total of 380 polymorphic EST-SSRs were used in the present study to develop the genetic linkage map and identify QTLs for anthracnose disease resistance.

## Materials and methods

### Mapping population

A mapping population of *D*. *alata* consisting of 94 F1 progenies developed from a cross of two diploid genotypes including TDa 95–310 (susceptible male parent) and TDa 95/00328 (moderately resistant female parent) was used in the study. Both parents are breeding lines developed at IITA and showed differential reactions to several isolates of *Colletotrichum gloeosporioides* [15]. Tubers of 96 yam genotypes, including both parents, were sliced into minisetts of 20–25 g and planted in plastic pots filled with sterilized soil. The experiment was carried out in the screenhouse for three months, an optimal age for vine cutting, and the vines were cut from each genotype to establish a new set of plants of uniform age and growth across all the genotypes before inoculation with the most virulent isolate of *C*. *gloeosporioides*, Kog01R1. The mini-tubers from each genotype were harvested that served as the planting materials of the following two years for assessment of anthracnose disease for a total of three years.

### Screening of mapping population for anthracnose disease

**Fig 1:**
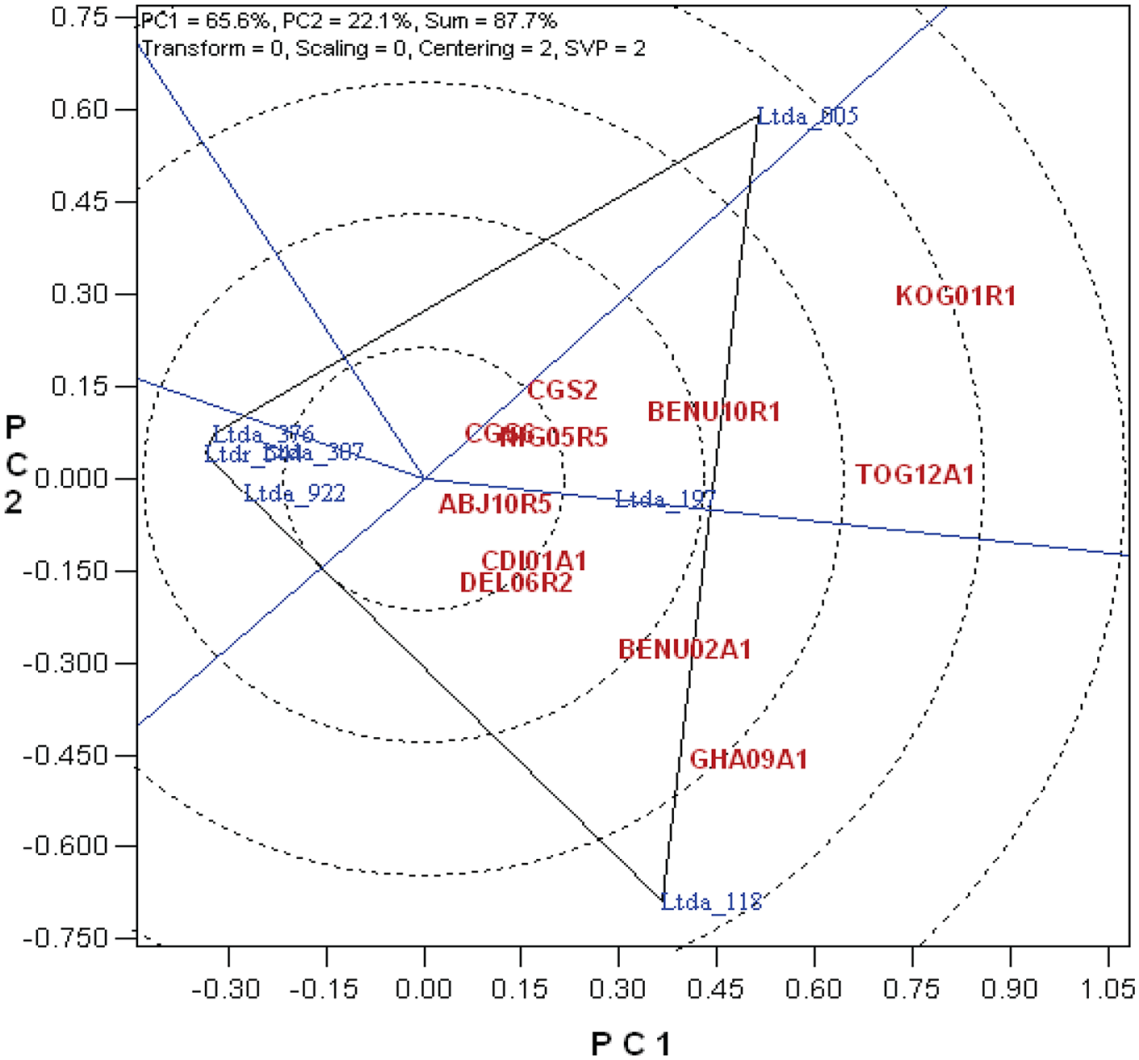
PCoA graph using anthracnose score data based on whole plant assay of 11 isolates of *C*. *gloeosporioides* on different *D*. *alata* varieties. The principle coordinate analysis (PCoA) graph indicates the virulence of 11 isolates when tested on different *D*. *alata* varieties based on detached leaf assay. Red: 11 *C*. *gloeosporioides* isolates; Blue: *D*. *alata* genotypes.

In a previous study, a total of 41 isolates of *C*. *gloeosporioides*, collected from naturally infected yam leaves from five West African countries (Nigeria, Ghana, Ivory Coast, Togo and Benin) were evaluated for virulence using several *D*. *alata* accessions [12, 15] using detached leaf assay in the laboratory using young fully-expanded leaves following the method of Green et al [26]. The detached leaves were scored 7 days after inoculation for anthracnose severity, based on proportion of affected leaf area at the point of inoculation, using a 1-to-5 rating scale, where 1 = 0 to 5, 2 = >5 to 10, 3 = >10 to 25, 4 = >25 to 50, and 5 = >50% of leaf area affected. Similarly, yam genotypes with anthracnose severity scores of 1, 2, 3, 4, and 5 were characterized as highly resistant, resistant, moderately resistant, susceptible, and highly susceptible, respectively. Based on the scores, three isolates—Kog01R1 (Nigeria), Tog12A1 (Togo), and Gha09A1 (Ghana)–were the most virulent (Fig 1) [16]. In the present study, the highly virulent fast-growing salmon (FGG) isolate of *C*. *gloeosporioides* (Kog01R1), was used for anthracnose screening. All 96 genotypes were planted in large plastic pots (18 cm in diameter and 25 cm in height) in screenhouse using a completely randomized design with four replications at International Institute of Tropical Agriculture (IITA; 07°30′01.7″ N, 003°54′29.4″ E), Ibadan, Nigeria during 2011, 2012 and 2013. The parents were considered as controlled checks in all three years’ evaluations. Whole-plant inoculation was used for disease-assessment and scoring [14, 27]. All the plants were spray-inoculated with spore suspension of Kog01R1 isolate (10^6^ spores mL –^1^) in the screenhouse using approximately 1 mL of inoculum per plant. The inoculated plants were scored for anthracnose severity from 3^rd^ day onwards until 11^th^ day on a scale of 1–5 using the percentage whole plant-area scoring method [25] with slight modification according to Onyeka et al [14]. Anthracnose score 1 represented high resistance with a leaf area damage of ≤ 2% and score 5 represented high susceptibility with a leaf area damage of ≥ 50%.

### DNA extraction

Genomic DNA from all 94 progenies and two parents was extracted from young leaf tissues collected before inoculation of the plants according to the procedures described by Sharma et al. [28]. DNA was extracted from 1 g of fresh leaves of 3-months-old screenhouse grown plants. DNA quality was assessed by running the samples on 0.8% agarose gel following electrophoresis, and the quantity was estimated on NanoDrop2000 spectrophotometer, using the ratio of absorbance at 260 nm and 280 nm to assess the concentration in ng/μl and purity level of the DNA.

### EST-SSR analysis

The DNA samples collected from the mapping population and the two parents were shipped to GBRU, USDA-ARS, Stoneville for genotyping. A total of 1,152 EST-SSRs were generated from >40,000 EST-sequences [23, 24] using SSRFinder software (http://www.maizemap.org/bioinformatics/SSRFINDER/README.ssrfinder) which located the SSRs in the EST-sequences. were tested for polymorphism on the two parents. A total of 584 EST-SSRs were polymorphic with 445 SSRs produced a single product (one locus), 70 with two products (possible indication of duplicates genes) and 69 were multi-allelic (possible indication of polyploidy). Based on quality of PCR products and allelic patterns, 380 SSRs were identified as good quality polymorphic SSRs [24]. A complete summary of the EST-SSRs, their forward and reverse sequences, repeat motifs, and product ranges are provided in Saski et al [24].

Primers were obtained from Integrated DNA Technologies (IDT, Coralville, IA) and normalized at 6 nmols in a 96-well plate. For genotyping, a tailing method was used to add a fluorescently labeled tag to the final PCR product as a third primer homologous to the tail. Forward primers were 5′ tailed with the sequence 5′-CAGTTTTCCCAGTCACGAC-3′ and the third tailed primer was Primer 5′-CAGTTTTCCCAGTCACGAC-3′ labeled with 6-carboxy-fluorescein. The reverse primers were tailed at the 5′ end with the sequence 5′-GTTT-3′ to promote non-template adenylation [29]. The 5 μl PCR reaction contained 10 ng genomic DNA, 0.4 μl Titanium Taq DNA Polymerase (Clontech, Mountain View, CA), 0.5 μl Titanium buffer, 0.04 μl dNTP (25mM), 0.8 μl FAM labeled primer (05 pmol/μl), 0.4 pmol of forward primer and 1.2 pmol reverse primer). The reaction was run on a BioRad thermocycler (Hercules, CA) at 95 °C for 1 min, 60 °C for 1 min (two cycles), 95 °C for 30 s, 60 °C for 30 s, 68 °C for 30 s (27 cycles), and a final extension at 68 °C for 4 min. Fluorescently labeled PCR fragments were analyzed on an ABI 3730XL DNA Analyzer with ROX 500 size standard and data-processed using GeneMapper Version 5.0 (all three from Applied Biosystems, Foster City, CA). Each primer pair was then used to genotype the mapping population progenies to determine the allele number and size for each primer.

### Linkage map construction and QTL mapping

The EST-SSR data was summarized across all individuals following a typical diploid Mendelian inheritance for an F_2_ population, considering the cross-pollinated heterozygous nature of yam and its maintenance over the years through clonal propagation [3]. All progenies and markers with >25% missing data were removed from statistical analysis. Phenotypic data on anthracnose disease severity was recorded as percent of whole plants showing disease incidence. An analysis of variance (ANOVA) of disease severity was performed across all three years using PROC MIXED in SAS version 9.3 [30] to estimate heritability for disease severity considering the year effect as a factor. Linkage map construction was performed using JoinMap v 3.0 [31] using the parameters set for F_2_ population. Initial assignment of markers to linkage groups was based on minimum independence LOD threshold of 3.5 for each marker pair while regression mapping method was used to build the linkage map. Recombination frequencies were converted to map genetic distances in centiMorgans (cM) using the Kosambi mapping function [32] to determine the linear order of the markers within a linkage group for this population.

QTL analyses was performed using R/qtl [33]. The datasets of the 94 progenies (marker data and anthracnose scoring data) combined with the linkage map were used to identify regions associated with anthracnose disease. The QTL analysis was performed for each year separately and also using mean data across the years for anthracnose disease. Haley-Knott regression method [34] and simple interval mapping (SIM) method with mapping step-size of 2 Centimorgan (cM), permutation of 1000 iterations, confidence limit of 95% and threshold method [35] at 1% significance were used to identify potential QTL. Only QTLs at or above the LOD threshold for a given year and average data were considered as significant. However, in cases where significant QTL was not observed with the LOD threshold, arbitrary LOD threshold was applied to identify minor QTLs. Simple Interval Mapping was performed for QTL analysis to capture the variation of each year and the average score across all the years. The linkage map and position of significant QTL were then drawn using Mapchart 2.3 software [36]. The additive QTL effect (a) and the proportion of phenotypic variance explained by QTL (r^2^) were estimated using R/qtl and breeding view software [37].

## Results

### Evaluation for anthracnose resistance

**Fig 2:**
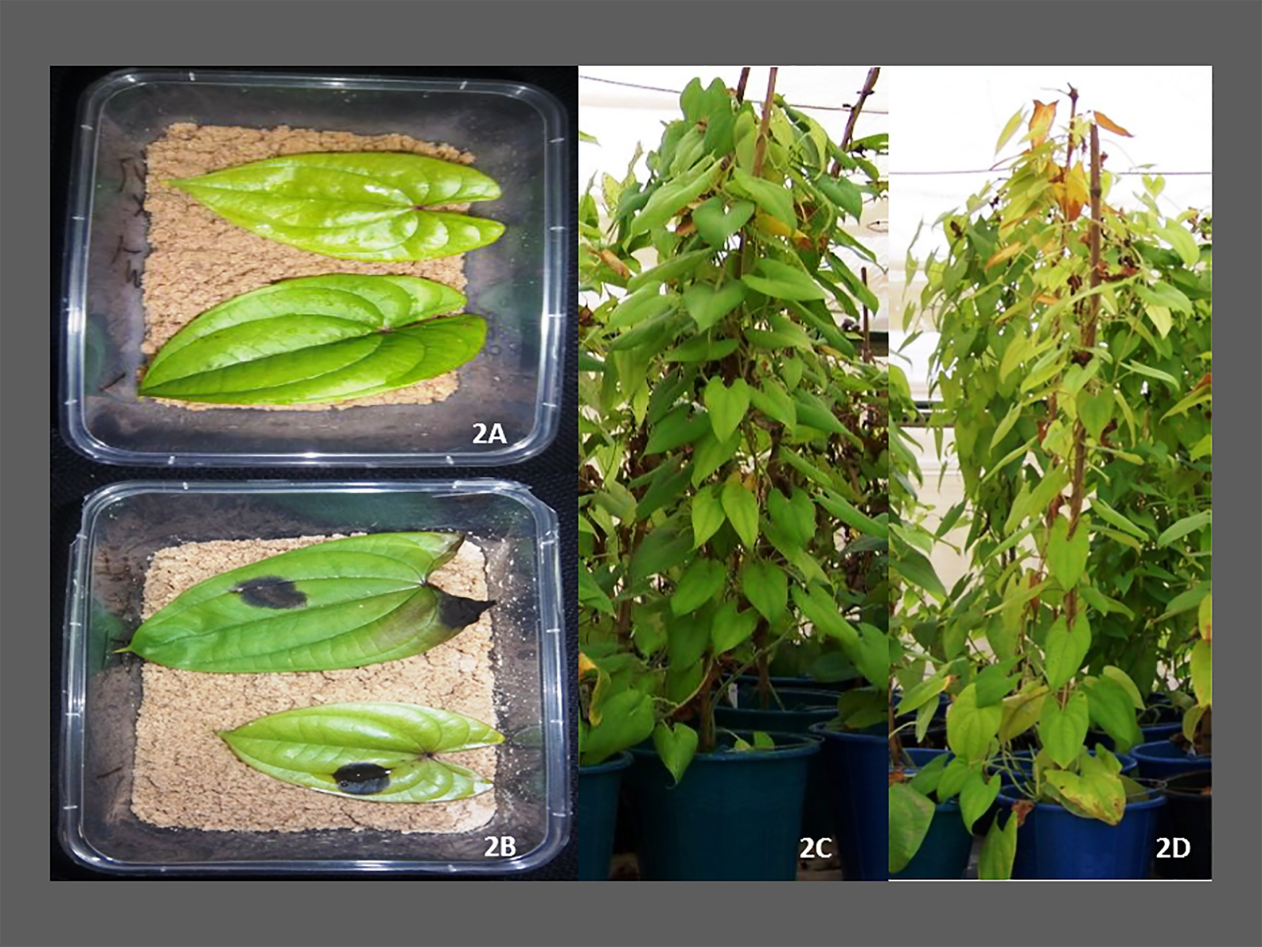
Characterization of *C*. *gloeosporioides* isolate Kog01R1 for virulence on two mapping population parents using detached leaf technique and whole plant inoculation. Phenotypic evaluation of two parents based on detached leaf assay in the lab and whole plant assay in the screenhouse. 2A and 2B: detached leaf assay on TDa 95/00328 and TDa 95–310 in the laboratory; 2C and 2D: whole plant assay on TDa 95/00328 and TDa 95–310 in the screenhouse.

A set of 41 *C*. *gloeosporioides* isolates collected from five different countries in West Africa was studied for their differences in causing anthracnose on different *D*. *alata* varieties using detached leaf technique and whole-plant inoculation [14, 26]. Eleven out of 41 isolates showed high virulence and a PCoA graph was used to visualize their virulence based on the distance from the centre (Fig 1). The first two principal coordinates accounted for 87.7% of total variation. Any isolate with eigenvalues >0.30 was considered as virulent, while those >0.60 as highly virulent. Based on this criterion, three isolates such as Kog01R1 (from Nigeria), Tog12A1 (from Togo) and Gha09A1 (from Ghana) showed high virulence and were grouped into highly virulent (Kog01R1) and virulent (Tog12A1 and Gh09A1) categories. These isolates also caused significant necrosis on the yam plants with Kog01R1 showing maximum virulence in both detached leaf and whole-plant inoculation techniques (Table 1, Fig 2). The highly virulent isolate Kog01R1 from Nigeria was selected to evaluate the mapping population consisting of ninety-four progenies. Kog01R1 has the features to exhibit high virulence at all stages of experiments carried out and it also showed the highest rate of infection in all the tested yam varieties, although there was variation in the rate of infection [14]. It is similar in characteristics to the fast- growing gray (FGG) isolates of *C*. *gloeosporioides*, and Abang et al [11] stated that FGG group is of high virulence as observed based on morphological characterization of isolates.

**Table 1:** Characterization of *C*. *gloeosporioides* isolates based on anthracnose virulence of the isolates on different yam varieties for detached leaf technique (DLA) and whole plant assay (WPA).

Isolates	TDa 99/00240		TDa 93–36		TDa 92.2		TDa 95/00005		Virulence
	DLA	WPA	DLA	WPA	DLA	WPA	DLA	WPA	
KOG01R1	4.7	3	3.1	3.5	3.2	3	2.5	3	Hv
GHA09A1	4.1	3.5	3	3	2.6	3	2	3	V
TOG12A1	3.9	2	2.8	3	2.4	3	2	2	V

DLA: Detached leaf assay; WPA: Whole plant assay; Anthracnose disease scoring on 1–5 scale with 1 = highly resistant; 2 = resistant; 3 = moderately resistant; 4 = susceptible; 5 = highly susceptible

**Fig 3:**
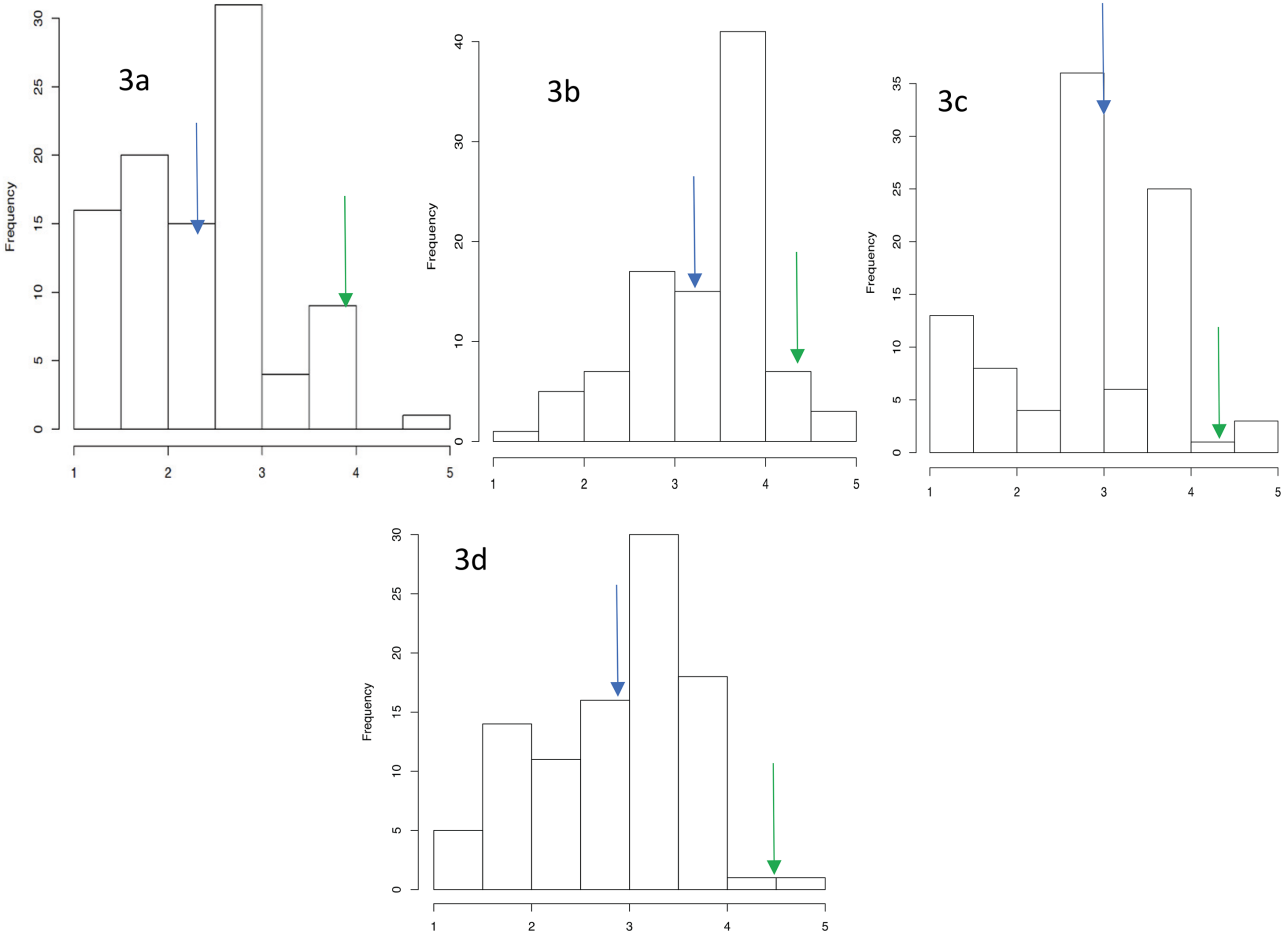
Mean phenotypic distribution of 94 progenies for anthracnose disease for individual years and average across three years. Frequency distribution of anthracnose disease among 94 progenies screened during 2011 (3a), 2012 (3b), 2013 (3c) and average across three years (3d). The arrows indicate the disease severity of the parental lines, TDa 95/00328 (Blue) and TDa 95–310 (Green).

The phenotypic evaluation of bi-parental population of 94 progenies for anthracnose disease was carried out for three years (2011, 2012 and 2013) in screenhouse using whole-plant inoculation technique. The parental clones showed a contrasting level of resistance. TDa 95/00328, moderately resistant female parent, showed a higher resistance (average score of 2.9) to the isolate than the highly susceptible male parent, TDa 95–310 (average score of 4.5). The phenotypic scoring of 94 progenies based on whole plant anthracnose severity resulted in 5.2% of highly resistant, 25.0% resistant, 44.8% moderately resistant, 24.0% susceptible, and 1.0% highly susceptible groups as their mean anthracnose incidence across three years (Fig 3) (S1 Table).

The phenotypic response of the mapping population to anthracnose disease appeared not to be influenced by different years as evident from ANOVA analysis (data not presented). The variance component for genotypes was 0.56 while that of phenotype was 0.61, suggesting that the variation for anthracnose disease among the mapping population progenies was mainly contributed by the genotypes. Broad-sense heritability (H^2^), which estimates the proportion of phenotypic variance that is due to genetic factors, was high (0.92).

### Linkage mapping

**Fig 4:**
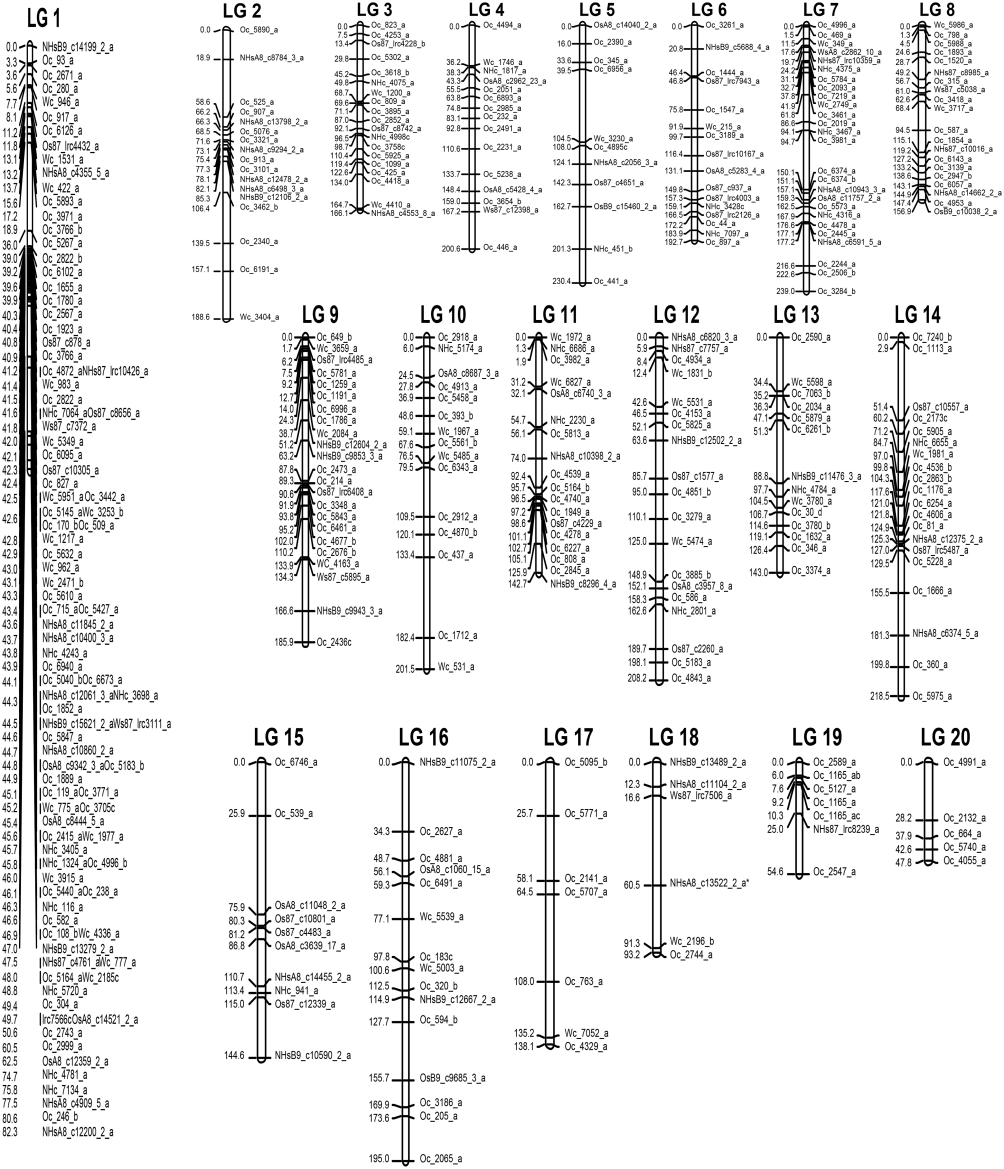
Genetic linkage map of water yam constructed using 380 EST-SSRs. Distribution of EST-SSRs across 20 linkage groups.

The genetic linkage map was constructed using 380 EST-SSR marker data on 94 segregating progenies (Fig 4). The final map comprised of all 380 markers mapped onto 20 linkage groups with a total length of 3229.5 cM and a LOD score of 3.5. Segregation distortion analysis revealed that majority (60.2%) of the markers segregating in the population followed a Mendelian segregation pattern of either an F1 (3:1) (8.5%) or F2 (1:2:1) (52%). Owing to the low number of markers used in the current study, the distorted one’s were not removed from the analysis and it did not affect their assignment to linkage groups and the highly distorted markers were mapped on linkage groups 7, 8 and 12. Linkage group 1 recorded the largest number of markers comprising of 97 markers with an average marker interval of 3.2cM. Linkage group 20 was the shortest with five markers (Table 2) (Fig 4). The number of EST-SSR markers mapped in each linkage group varied from 97 markers in LG 1 to 5 markers in LG 20, with an average of 19 EST-SSRs per linkage group (Table 2). The average distance between markers was 14.2 cM across 20 linkage groups, with largest distance between two markers of 17.9 cM and 26.4 cM in LG 5 and LG 17, respectively.

**Table 2:** Length, number of markers, and median distance between markers for each linkage group.

Chromosome	Length (cM)	Number of Marker	Mean distance between markers (cM)	Number of long gaps (>10 or 20 cM)
**1**	82.3	97	0.2	3.1
**2**	188.6	17	3.6	37.7
**3**	166.1	19	6.7	25.9
**4**	200.6	15	10.7	35.7
**5**	230.4	11	17.9	65.1
**6**	192.7	16	11.7	28.2
**7**	239	26	5.4	43.4
**8**	156.9	20	5.4	23.6
**9**	185.9	23	4	27.7
**10**	201.5	15	10.6	45.2
**11**	142.7	18	2.4	26.9
**12**	208.2	19	8.8	29.0
**13**	143	14	7.3	37.1
**14**	218.5	20	8.8	38.3
**15**	144.6	10	5.6	51.0
**16**	195	15	13.5	33.0
**17**	138.1	7	26.4	45.7
**18**	93.2	6	12.3	47.1
**19**	54.6	7	3.8	32.5
**20**	47.8	5	7.4	33.7
**Average**	**161.5**	**19**	**14.2**	**33.8**

### QTL analysis

**Fig 5:**
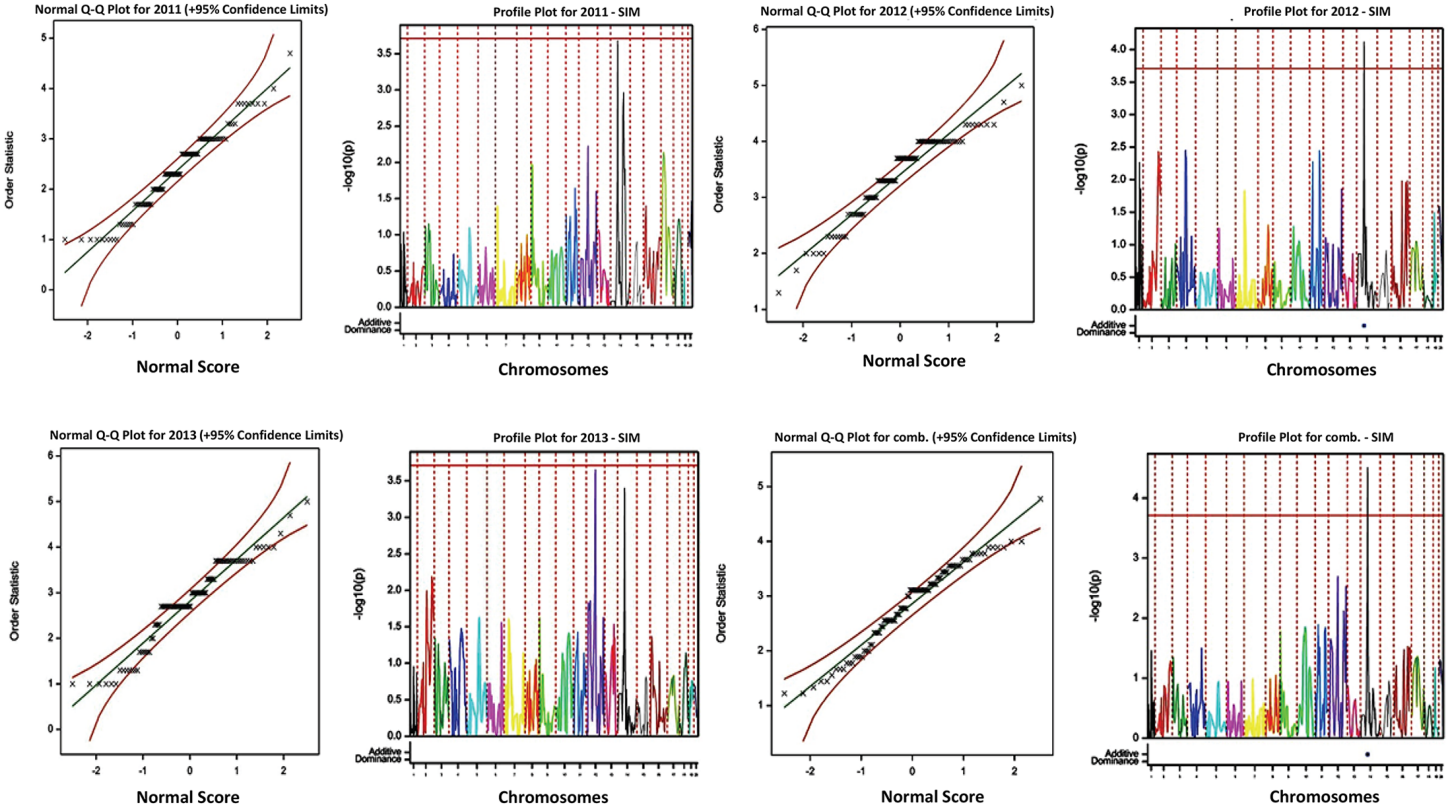
QTLs for anthracnose disease based on average score across three years and SSR data at threshold LOD score using simple interval mapping (SIM). Distribution of QTL associated with anthracnose disease. The bar represents the threshold LOD confidence for the QTL. QTL effects: Additive (Blue: Parent 1, Yellow: Parent 2); Dominance (Blue: Negative, Yellow-Red: Positive).

The *C*. *gloeosporioides* isolate used in the present study was highly virulent but had differing responses on both parents and the progenies showing segregation pattern ranging from high resistance with no visual symptoms (average disease severity score of 1.5) to high susceptibility (average disease severity score of 4.5) over three years. The distribution of disease scores for individual three years and averaged over three years for 94 segregating progenies was skewed towards resistance with majority of the progenies falling under resistance or moderately resistance category (1: highly resistant, 2: resistant, 3: moderately resistant, 4: susceptible, and 5: highly susceptible) (Fig 3). QTL analysis was carried out using disease severity score data collected during individual years and averaged over three years and SSR data on 94 progenies. In the combined analysis using average mean disease scores across years, a significant QTL was observed on LG 14 at position 77.9 cM (between 71.1–84.8 cM) at a LOD score of 4.5 explaining 68.9% of total phenotypic variation (Table 3) (Fig 5). No significant QTL was found for anthracnose disease in 2011 at threshold LOD score, however, at a lower LOD threshold of 3.6, a significant QTL was observed on LG 14 in the same position (Table 3; Fig 5). Similarly, for 2012, one significant QTL was observed on LG 14 at position 77.9 cM (between 71.1–84.8 cM) explaining 75.6% of phenotypic variation. For 2013, two significant QTL was observed, one on LG 12 and another on LG 14 (Fig 5). The QTL on LG14 was consistently and significantly associated with anthracnose resistance at threshold significant LOD score in two out of three years and average score data. This QTL was consistently found at position 77.9 cM falling between 71.1–84.8 cM (Table 3). Additional minor QTLs (LOD ≥ 2.5) were observed on LG 1 and 4 for 2012 at LOD threshold of 3.1 and 2.9, respectively; while QTLs on LG 9 and 16 for 2013 at LOD of 2.7 and 2.5, respectively. For average over three years, an additional QTL on LG12 was observed at LOD score of >2.5 (Fig 5). Similarly, for average data, an additional QTL was observed on LG 12 at a LOD threshold of 2.9 (Fig 5; Table 3). For the consistent QTL on LG 14, the female parent TDa95/00328 (moderately resistant to anthracnose disease) positively contributed the allele conferring anthracnose disease resistance, while remaining minor QTLs are associated with alleles from both parents wherein the female parent contributed positively towards anthracnose resistance.

**Table 3:** Summary of QTL analysis with significant QTLs identified in different years as well as the average over three years.

QTL location (cM)	Linkage group	Marker interval	Position interval (cM)	LOD	Pvalue (F)	Percent variation explained	Contributing parent
**2011**							
14@77.9	14	Os87_c10557_a -Oc_1666_a	71.1–84.8	3.6	0.025	30.8	TDa95/00328
**2012**							
1@45.1	1	Oc_917_a-Oc_913a	8.1–75.4	3.1	0.037	12.23	TDa95/00328 and TDa95-310
4@92.8	4	Oc_232_a-Oc_2231_a	83.1–110.6	2.9	0.035	9.23	TDa95/00328 and TDa95-310
14@77.9	14	Os87_c10557 a Oc_1666_a	71.1–84.8	4.1	0.0003	75.6	TDa95/00328
**2013**							
9@45.9	9	Os87_lrc4485_a Wc_531_a	6.2–51.2	2.7	0.2	5.2	TDa95/00328 and TDa95-310
12@92.5	12	Wc_1831_b—Oc_4843_a	12.4–208.2	3.8	0.002	39.6	TDa95/00328
14@77.9	14	Os87_c10557 a Oc_1666_a	71.1–84.8	3.7	0.0003	33.4	TDa95/00328
16@100.6	16	NHsB9_c11075_2_a—Oc_2065_a	0–195.0	2.5	0.009	5.21	TDa95/00328 and TDa95-310
**Average across 3 years**							
12@167.5	12	Wc_1831_b—Oc_4843_a	12.4–208.2	2.9	0.003	4.9	TDa95/00328 and TDa95-310
14@77.9	14	Os87_c10557 a Oc_1666_a	71.1–84.8	4.5	0.0004	68.9	TDa95/00328

## Discussion

The availability of a saturated genetic linkage map is a valuable tool for genetic research and molecular breeding. The genetic linkage map developed in this study is the first *D*. *alata* genetic map constructed using EST-SSRs that spanned a total length of 3229.5 cM and included 20 linkage groups. The earlier genetic maps on *D*. *alata* were based on AFLPs [20, 22] that covered 1538 cM and 1233 cM, respectively and included 20 linkage groups. Petro et al [20] estimated the total length of yam genome to be 1923 cM, however, it is estimated that the genome size of *D*. *alata* is 534 Mb (Jessica B. Lyons, *personal communication*), which is almost similar to that of *D*. *rotundata* (540 Mb) [23]. A comparative analysis to annotate the EST-sequences from *D*. *alata* with the scaffold sequences of *D*. *rotundata* showed no collinearity, and this may be attributed to the diverse center of origin or crossing incompatibility among these two species [38]. Till date, there is no report on successful hybridization between *D*. *rotundata* and *D*. *alata* (Robert Asiedu, *personal communication*). The genetic map produced in the present study is sparsely saturated with random distribution of EST-SSRs across different linkage groups and it is expected that with the availability of more markers (SSRs or SNPs), a dense consensus map can be generated with appropriate positioning of markers in each linkage group.

The observed segregation distortion among the markers was 39.8%, and this may be due to small size of the mapping population (94 individuals) or genotyping errors or biological factors such as gametic and/or zygotic selection or it could be due to physiological and environmental factors [39, 40, 41]. Similar level of distortion was also observed by Petro et al [20] while constructing the linkage map from an intraspecific F1 population in *D*. *alata*.

The most virulent isolate of *C*. *gloeosporioides*, Kog01R1, was used to infect both parents in all the three years (2011, 2012 and 2013). The severity of symptoms was much higher on TDa 95–310 (highly susceptible parent) than on TDa 95/00328 (moderately resistant parent) using both whole plant assay and detached leaf assay. The segregation of 94 progenies for anthracnose response showed a distribution from highly resistance to highly susceptible (Fig 3) although it was skewed towards resistance with higher number of progenies falling within moderately resistant and resistant category. This is in support of the findings of Petro et al [20] and Mignouna et al [22] which described that resistance to anthracnose disease is dominantly and quantitatively inherited. This is also confirmed with high level of heritability (0.92) observed in the current study.

This study identified a consistent QTL against most virulent isolate of *C*. *gloeosporioides*, however, the resistance of *D*. *alata* to anthracnose disease could be isolate-specific or non-specific [20]. There is a need to use combination of two or more isolates for co-infection, to validate the isolate-specificity and non-specificity for resistance, and to understand the isolate-isolate interaction and host-isolate interaction causing anthracnose disease. Use of QTL-approach to identify isolate specificity for quantitative disease resistance has been reported in several studies [42, 43, 44]. So far, two studies have been undertaken for mapping QTLs controlling resistance or partial resistance to anthracnose disease in water yam [20, 22]. Both studies used AFLP maps and detected one [22] and nine QTLs [20], respectively for partial resistance to anthracnose disease explaining 10% and 26.4 to 73.7% of phenotypic variance. However, the non-availability of SSR markers and common linkage group nomenclature in both maps makes it difficult to compare the location of detected QTLs in these two studies. In the present study, we used the same resistant female parent, TDa 95/00328 as used by Mignouna et al [22] and EST-SSRs to develop the linkage map and identify the QTLs for anthracnose resistance. We detected one consistent QTL on linkage group 14 across different years of evaluation and average over years that explained 68.9% of the phenotypic variance in the mapping population progenies, indicating the presence of a major QTL in the region. However, this QTL is based on a small number of segregating population (94 progenies) derived from a cross between moderately-resistant and susceptible parents. The small population size used in QTL mapping is likely to reduce the power of QTL significance tests, thus underestimating the number of QTLs involved in a trait. Simultaneously, the QTL effects with small progeny sizes are also overestimated [44]. There is also need for using diverse parents including highly resistant and highly susceptible parental genotypes for anthracnose disease to generate the segregating population and capture a broader range of diversity for the trait. Similar study can also be carried out using next generation sequencing techniques to develop SNP-based dense genetic linkage map to validate the QTL reported in the present study and identify additional major QTLs for this devastating disease. This will further allow to develop SNP-chip for marker assisted selection especially at early generation and reduce the breeding cycle of the crop, which currently takes about 4–5 years.

## Conclusions

The linkage map obtained in this study showed a good coverage of the expected genome size of water yam (*Dioscorea alata*). This is the first study to report the use of EST-SSRs to generate a genetic linkage map and detection of QTLs using the most virulent isolate of *C*. *gloeosporioides* prevalent in West Africa. This has provided new opportunities to construct dense linkage map and validate the QTLs and understanding the host-pathogen interaction for anthracnose disease in this crop. Overall, a significant portion of the phenotypic variation remained undetected in this study and a large number of minor-effect QTL could not be detected with significant LOD thresholds. Given the quantitative nature of the trait, future studies should target larger mapping populations involving diverse genetic backgrounds with high level of resistance, robust and precise disease phenotyping across wide environments, and next-generation sequencing techniques to identify a broad spectrum of QTLs conferring anthracnose resistance in *D*. *alata*. Studies are underway with additional mapping populations to test this hypothesis and generate a high-resolution genetic and physical map of *D*. *alata*. This will further elucidate the application of marker-assisted selection for anthracnose disease resistance in this important staple food crop.

## Supporting information

S1 Table Anthracnose disease scoring data in 2011, 2012, 2013 and average across three years based on whole plant assay. (XLSX)
